# Nutritional status and risk factors for stunting in preschool children in Bhutan

**DOI:** 10.1111/mcn.12653

**Published:** 2018-11-09

**Authors:** Yunhee Kang, Víctor M. Aguayo, Rebecca K. Campbell, Laigden Dzed, Vandana Joshi, Jillian L. Waid, Suvadra Datta Gupta, Nancy J. Haselow, Keith P. West

**Affiliations:** ^1^ Center for Human Nutrition Johns Hopkins School of Public Health Baltimore MD; ^2^ UNICEF New York NY; ^3^ Ministry of Health Royal Government of Bhutan Thimphu Bhutan; ^4^ UNICEF Bhutan Thimphu Bhutan; ^5^ Helen Keller International Dhaka Bangladesh; ^6^ Asia Pacific Regional Office Helen Keller International Phnom Penh Cambodia

**Keywords:** Bhutan, childhood stunting, nutritional trend, risk factors, South Asia, wasting

## Abstract

Childhood malnutrition remains endemic in South Asia, although the burden varies by country. We examined the anthropometric status and risk factors for malnutrition among children aged 0–59 months through the 2015 National Nutrition Survey in Bhutan. We assessed in 1,506 children nutritional status (by *z*‐scores of height‐for‐age [HAZ], weight‐for‐height [WHZ], and weight‐for‐age [WAZ]), estimating prevalence, adjusted for survey design, of stunting, wasting, underweight, and overweight (<−2 for HAZ, WHZ, and WAZ and >2 for WHZ). Children were also assessed for pedal oedema. We conducted multivariable linear/logistic regression analysis to identify child, maternal, and household risk factors for childhood undernutrition and overweight, excluding children with oedema (1.7%). Mean (SE) HAZ, WHZ, and WAZ were −0.82 (0.13), 0.10 (0.04), and −0.42 (0.05), respectively. Prevalence of stunting, wasting, underweight, and overweight were 21.2%, 2.6%, 7.4%, and 2.6%, respectively. In multivariable regressions, risk of stunting significantly increased by age: 5.3% at <6 months (reference), 16.8% at 6–23 months (OR = 3.06, 95% CI [0.63, 14.8]), and 25.0% at 24–59 months (OR = 5.07, [1.16, 22.2]). Risk of stunting also decreased in a dose–response manner with improved maternal education. None of the examined variables were significantly associated with wasting or overweight. Despite a WHZ distribution comparable with the World Health Organization reference (with ~2.6% vs. an expected 2.5% of children beyond 2 z in each tail), stunting persists in one fifth of preschool Bhutanese children, suggesting that other nutrient deficits or nonnutritional factors may be constraining linear growth for a substantial proportion of children.

**Key messages**

- Despite a steady decrease in undernutrition since 1986, stunting persists among one‐fifth of under‐five children in Bhutan.
- The risk of preschool Bhutanese children being stunted increases with age, which may be partly mitigated by improved maternal education.
- Weight for height of Bhutanese children, however, tracks the WHO child growth reference, suggesting that factors other than energy deficit may be limiting normal linear growth achievement.
- Additional risk factors of stunting need to be understood and considered in the design of nutrition and other interventions intended to reduce stunting in the presence of apparently adequate weight for height status of children.

## 1 INTRODUCTION

Stunting remains a chronic public health and development problem in South Asia, where 40%, or 58.7 million, of the world's stunted preschool‐aged children reside (United Nations Children's Fund [UNICEF], World Health Organization [WHO], & World Bank Group, 2018). Notwithstanding, there has been a decline in prevalence of stunting from 49.6% in 2000 to 33.3% in 2017 (UNICEF et al., 2018), attributed to many factors, including, poverty reduction, women's education, access to improved sanitation and health and nutrition programs (Headey & Hoddinott, 2015; Headey, Hoddinott, & Park, 2016; Headey, Hoddinotta, Ali, Tesfaye, & Dereje, 2015), although socio‐economic inequalities that may underlie stunting persist in many countries (Krishna, Mejía‐Guevara, McGovern, Aguayo, & Subramanian, 2018; Restrepo‐Mendez, Barros, Black, & Victora, 2015).

Childhood stunting may result in short‐ and long‐term adverse consequences such as increased childhood morbidity and mortality, impaired cognitive development, increased risk of obstetric complications and mortality in women of reproductive age, reduced productivity and earnings in adulthood, and intergenerational health and nutrition effects (Black et al., 2013). Accelerating the reduction of stunting in South Asia is central to the achievement of the Global Nutrition Targets of reducing by 40% the number of stunted children under 5 years old by 2025 (WHO, 2014) endorsed by 2030 Agenda for Sustainable Development (http://www.un.org/sustainabledevelopment/development-agenda).

Bhutan is a landlocked country in the eastern Himalayas with a projected population of 779,666 in 2017 (Bhutan National Statistics Bureau, 2017). The current prevalence of stunting among preschool children in Bhutan (21.2%) is lower than in neighbouring South Asian countries (Nutrition Program, Department of Public Health, Ministry of Health, 2016): Bangladesh (36%; Bangladesh National Institute of Population Research and Training, Mitra and Associates, & ICF International, 2016), India (39%; UNICEF, 2016), or Nepal (37%; Nepal Central Bureau of Statistics, 2015). Apart from international efforts towards achieving the Millennium Development Goals (http://www.un.org/millenniumgoals), this lower rate of stunting may be due in part to the government of Bhutan's almost four decade national initiative called the Gross National Happiness (http://www.grossnationalhappiness.com/). Implemented since 1979, this initiative achieved remarkably positive health, nutrition and development outcomes and a rapid and inclusive poverty reduction with an average economic growth of 6.7% between 2009 and 2013 (National Statistics Bureau Royal Government of Bhutan & World Bank, 2017). Notably, the national poverty rate was reduced by one third from 23.2% in 2007 to 8.2% in 2017 (National Statistics Bureau Royal Government of Bhutan & World Bank, 2017).

Some South Asian countries such as Bangladesh or India have more established nutrition surveillance and research programs on which national policy and programs can be planned and evaluated. However, only a few national survey reports and studies using nationally representative data are available to provide information on maternal and child nutrition in Bhutan (Aguayo, Badgaiyan, & Paintal, 2015; Zangmo, de Onis, & Dorji, 2012).

Against this backdrop, our study provided in‐depth information on the nutritional status of Bhutanese children using data from the National Nutrition Survey (NNS) 2015 (Nutrition Program, Department of Public Health, Ministry of Health, 2016). The objective of this study is threefold: first, we describe the nutritional status of children (i.e., *z*‐scores, stunting, wasting and overweight) by geographic and socioeconomic characteristics. Secondly, we identify the child‐, maternal‐ and household‐level predictors of nutritional outcomes of interest. Third, we describe time trends of nutritional status across prior surveys from 1986 to 2015.

## 2 PARTICIPANTS AND METHODS

### 2.1 Data sources

We used NNS 2015 as the primary source of data for this study. Further, we used NNS 1986–1988 (first; Directorate of Health Services, 1989), NNS 1999 (second; Namgyal & Yoezer, 1999), NNS 2008 (third), and the Multiple Indicator Cluster Survey (MICS) 2010 to provide additional information about the time trends in childhood malnutrition in the country.

### 2.2 Study design, sampling, and sample size

The study design, sampling methods, and main findings of the NNS 2015 are presented in detail elsewhere (Nutrition Program, Department of Public Health, Ministry of Health, 2016). Briefly, the country is divided geographically into western, central, and eastern regions and administratively into 20 Dzongkhags (districts). NNS respondents were selected with a national multistage sampling method. Two Dzongkhags were selected from each region with probability proportional to size sampling. Punakha and Lhuntse, estimated before the survey to be the highest and lowest performing Dzongkhags, respectively, were also included. Within each selected Dzongkhag, subareas were selected with probability proportional to size sampling and with a target urban/rural balance reflecting the population distribution in each region. In each area, 12 households were selected using systematic random sampling, for a total of 3,571 households surveyed. Our study used data, including anthropometric measures, for a total of 1,506 children aged 0 to 59 months identified in the surveyed households.

#### 2.2.1 Available variables

The NNS 2015 collected data using survey modules relevant to assessing household‐, mother‐ and child‐level variables, including children's anthropometry (weight and length at <2 years and weight and height at older ages), which was measured followed standard procedures (Nutrition Program, Department of Public Health, Ministry of Health, 2016). In addition, children were assessed for presence of pitting pedal oedema in both feet as a separate clinical indicator for severe acute malnutrition. Anthropometric *z*‐scores and the prevalence of undernutrition were calculated from child length/height (*n* = 1,481) and weight (*n* = 1,490), excluding missing or values outside biologically reasonable ranges, based on the WHO reference values (WHO Multicentre Growth Reference Study Group, 2006).

The prevalence of stunting, wasting, underweight, or overweight was defined as the proportion of children whose height‐for‐age (HAZ), weight‐for‐height (WHZ), and weight‐for‐age (WAZ) scores were more than two standard deviations below the median of the population standard (or above the referent median of WHZ; for overweight; WHO Multicentre Growth Reference Study Group, 2006). Factors associated with nutritional outcomes were considered at household, maternal and child levels based on UNICEF's conceptual framework of undernutrition (UNICEF, 1997). Household characteristics included household size, land ownership, livestock ownership, type of house wall materials, perceptions of household food insecurity, food consumption score (FCS), household water source, water storage and treatment, sanitation facilities and receipt of any benefits from government programs. Maternal characteristics included mother's age and education level. Child characteristics included sex and age. Characteristics at all levels were grouped into appropriate categories. A household wealth index was developed by the National Statistics Bureau and used for the NNS 2015 report, using principal component analysis on household asset variables. Perception of food insecurity was a composite variable of food insecurity that household experienced any out of four cases for the one month preceding the survey: (a) worry about not enough foods, (b) eat only rice/kharang/flour, (c) eat a smaller amount/skip meals at any meal time, and (d) eat fewer meals in a day (Nutrition Program, Department of Public Health, Ministry of Health, 2016). The FCS is defined as “a composite score based on dietary diversity, food frequency, and relative nutritional importance of different food groups in the past 7 days” (World Food Programme, 2008). Based on FCS cut‐off criteria, our study divided the households into two categories: acceptable or borderline/poor.

#### 2.2.2 Statistical analysis

Stata version 14 (StataCorp LP, College Station, TX, USA) was used for all statistical analyses. The prevalence of undernutrition and distribution of *z*‐scores were presented by region, rural/urban, household socioeconomic status, maternal education and child age and sex. Data analysis was conducted in two phases. First, univariate linear or logistic regression was conducted to identify variables that were associated with *z*‐scores and degrees of stunting (<−1 HAZ, <−2 HAZ, and <−3 HAZ), wasting (<−2 WLZ), overweight (>2 WLZ) and underweight (<−2 WAZ). Variables associated with each outcome (*P* < 0.10) were included in multivariable regression models. We also adjusted for child age and sex, household wealth quintile and type of sanitation facility used (i.e., improved vs. not) as contextual covariates in multivariable models for stunting, although they were not significant in the univariate analysis (Stewart, Iannotti, Dewey, Michaelsen, & Onyango, 2013). A variance inflation factor was checked for each univariate predictor to assess whether the predictors were highly correlated, and all tested variables reported variance inflation factors less than 3.0. Variables that kept significant association (*P* value < 0.05 or judged by 95% CI for odds ratios) in the multivariable regression were considered as associates of nutritional status indicator *z*‐scores or event of undernutrition or overweight. The sampling design of the original survey was taken into account in all analyses by using “svy” commands to estimate summary statistics at the population level and to evaluate characteristics associated with nutritional status.

### 2.3 Ethical approval

Approval for the NNS 2015 methodology and sampling strategy was obtained from the National Statistical Bureau of Bhutan, and ethical clearance for the study was sought from the Research Ethical Board of Health, Ministry of Health. This secondary data analysis was deemed exempt from ethics review by the Institutional Review Board, Johns Hopkins School of Public Health.

## 3 RESULTS

### 3.1 Selected household, maternal and child characteristics

Out of 1,506 children aged 0 to 59 months, 51.8% were female and the mean age was 29.9 (SE: 1.0) months, 41.7% were from the western, 23.7% were from the central, and 34.6% were from the eastern regions (Table 1), in line with the population of each of these regions. Most households (84.3%) had access to improved drinking water sources, stored water in a container (81.6%), and treated (sanitized) water (90.1%). Seven out of 10 households (68.3%) had improved sanitation facilities. The proportion of households reporting to be food insecure was low (2.3%). The majority of households (93.7%) showed acceptable FCS. Twenty‐four percent of all households reported receiving benefits from any type of government programs. Half of mothers of surveyed children had either no education or informal education only (35.4% and 15.4%, respectively), and 35.6% had completed high school or higher education.

**Table 1:** Characteristics of households, mothers, and children aged 0 to 59 months from the National Nutrition Survey (NNS) 2015

	National (*N* = 1,506)	Region			Area	
		West (*n* = 645)	Central (*n* = 316)	East (*n* = 545)	Urban (*n* = 317)	Rural (*n* = 1,189)
Household level						
Region, %						
West	41.7^a^	—	—	—	40.0	43.3
Central	23.7	—	—	—	22.3	25.0
East	34.6	—	—	—	37.7	31.7
Location, %						
Urban	49.0	47.0	46.2	53.3	—	—
Rural	51.0	53.0	53.8	46.7	—	—
Wealth index quintiles,^b^ %						
Lowest	21.2	21.6	14.0	25.6	0.0	41.5
Low	18.8	19.7	23.9	14.2	5.7	31.3
Medium	21.7	16.4	23.0	27.3	29.0	14.8
High	20.9	18.5	21.8	23.2	33.6	8.8
Highest	17.4	23.8	17.3	9.7	31.7	3.6
Wall materials, %						
Mud/wood	52.1	56.6	50.5	47.8	22.8	80.2
Cement/concrete	47.9	43.4	49.5	52.2	77.2	19.8
Owns land, %	63.4	64.4	74.6	54.6	40.9	85.0
Owns any livestock, %	40.9	40.7	47.8	36.4	4.2	76.4
Improved water source,^c^ %	84.3	83.7	89.7	81.2	84.6	83.9
Stored water in a container, %	81.6	78.0	79.8	87.1	81.8	81.4
Water treatment,^d^ %	90.1	91.1	92.4	87.5	99.4	81.2
Improved sanitation, %	68.3	66.6	68.8	69.9	77.5	59.4
Food insecure,^e^ %	2.3	3.3	1.9	1.4	0.6	3.9
Acceptable food consumption score (FCS),^f^ %	93.7	94.5	94.7	92.2	96.1	91.5
Benefited from government programmes, %	23.7	27.5	26.7	17.3	4.1	42.8
Household size ≥6, %	37.8	44.4	38.2	29.7	25.7	49.5
Maternal level						
Age, years, mean (SE)	29.3 (0.5)	29.7 (1.0)	29.4 (0.9)	28.7 (0.4)	29.5 (0.4)	29.0 (0.6)
<30 years	55.6	52.9	58.7	56.7	53.2	58.0
30–40 years	36.1	37.6	31.3	37.8	39.9	32.6
≥41 years	8.2	9.5	10.0	5.5	7.0	9.4
Education, %						
*n*	(1,430)	(609)	(297)	(524)	(300)	(1,130)
College	6.7	9.2	4.7	5.2	12.3	1.3
High school	29.0	29.6	33.3	26.2	43.6	15.6
Primary	13.5	18.6	12.5	8.3	9.4	17.5
None	35.1	34.9	35.0	35.5	25.5	44.4
Informal	15.3	7.7	14.4	24.9	9.2	21.2
Child level						
Sex, %						
Female	51.8	55.0	53.6	46.6	49.3	54.1
Male	48.2	45.0	46.4	53.4	50.7	45.9
Age, months, mean (SE)	29.9 (1.0)	29.5 (1.9)	31.2 (0.6)	29.6 (1.3)	29.9 (1.6)	29.9 (0.8)
0–5 months, %	8.4	9.6	4.9	9.5	10.5	6.5
6–23 months, %	30.2	32.9	29.7	27.3	26.1	34.1
24–59 months, %	61.4	57.5	65.5	63.2	63.4	59.4

*Note*. Missing values: maternal education, *n* = 76; maternal age, *n* = 45.

a Sampling design of the original survey was taken into account in all analyses by using “svy” commands to estimate summary statistics at the population level.

b Household asset score generated with principal component analysis as was done in the original analysis of the National Nutrition Survey data (Nutrition Program, Department of Public Health, Ministry of Health, 2016) but restricted to households with a child <5 years old who participated in the anthropometry assessment. The included variables were type of water source, shared sanitation facilities, toilet facilities, floor materials, roof materials, wall materials, cooking fuel, ownership of land, ownership of livestock, land of orchard, dry land, wet land, number of rooms, ownership of livestock, type of livestock (cattle/buffalo/yaks, pigs, horses, goats/sheep, poultry), sofa set, electric iron, bukhari, rice cooker, curry cooker, refrigerator, modern stove, water boiler, microwave oven, bicycle, tractor, power tiller, jewellery, motorbike/scooter, sechu gho/kira, family car, other vehicle, washing machine, sewing machine, television, VCR/VCD/DVD, grinding machine, wrist watch and weaving tool.

c Bhutan‐specific definition of household access to an improved water source is piped water into the household only.

d In the NNS 2015 survey, mothers were asked if she adds anything to the water to make it safer to drink.

e Food insecurity was one or more affirmative answers to the series of food security questions, represented as a composite variable of food insecurity that household experienced any out of four cases in the last month: (a) worry about not enough foods, (b) eat only rice/kharang/flour, (c) eat a smaller amount/skip meals at any meal time and (f) eat fewer meals in a day.

f Food consumption score (FCS) was based on household dietary diversity, food frequency and relative nutritional importance of different food groups in the past 7 days (World Food Programme, 2008).

### 3.2 Clinical oedema

Among the 1,506 children, 26 (1.7%) were classified with pedal oedema and included in the nutritional analysis in the NNS 2015 (Nutrition Program, Department of Public Health, Ministry of Health, 2016). Although these children were comparable with others in HAZ, oedema can distort WHZ and WAZ status; thus, oedematous children were excluded from anthropometric classification and indicator summaries in our analysis.

### 3.3 Anthropometric status

Among the 1,480 nonoedematous children, mean (SE) WHZ was 0.10 (0.04), HAZ was −0.82 (0.13), and WAZ was −0.42 (0.05). The prevalence of wasting (<−2 WHZ) was 2.6%, comparable for both sexes (~2.6%), all regions (2.1% to 3.2%) and urban (2.0%) and rural (3.1%) areas (Table 2 and Figure S1), although was virtually nil among children whose mothers had a college degree **(**Table S2). A similar national prevalence was observed for overweight (>2 WHZ; 2.6%), also comparable by sex (1.9% [female] to 3.4% [male]), region (1.5% to 3.4%) and urban/rural setting (~2.7%; Table 2, Figure S2), but showing no trend with maternal education. There were no trends in prevalence of wasting or overweight by household wealth (Table S2).

**Table 2:** Nutritional status of children aged 0 to 59 months by region and area from the National Nutrition Survey 2015

	National^a^	Region			Area	
		West	Central	East	Urban	Rural
(*n*)	1,414	600	302	512	296	1,118
HAZ, mean (SE)	−0.82 (0.13)^b^	−0.72 (0.28)	−0.63 (0.07)	−1.06 (0.16)	−0.57 (0.13)	−1.05 (0.11)
<−1, % (SE)	48.0 (0.04)	45.0 (0.10)	44.8 (0.02)	53.9 (0.04)	40.7 (0.05)	55.0 (0.04)
<−2, % (SE)	21.2 (0.02)	16.3 (0.03)	18.7 (0.04)	28.8 (0.03)	16.2 (0.02)	26.0 (0.02)
<−3, % (SE)	6.2 (0.01)	4.7 (0.02)	6.9 (0.02)	7.5 (0.02)	3.5 (0.01)	8.9 (0.01)
*n*	1,433	609	304	520	299	1,134
WHZ, mean (SE)	0.10 (0.04)	0.12 (0.05)	−0.02 (0.05)	0.17 (0.09)	0.09 (0.06)	0.11 (0.05)
<−2, % (SE)	2.6 (0.01)	2.1 (0.00)	3.2 (0.01)	2.7 (0.01)	2.0 (0.01)	3.1 (0.01)
<−3, % (SE)	0.4 (0.00)	0.8 (0.00)	0.2 (0.00)	0 (0.00)	0.3 (0.00)	0.5 (0.00)
>2, % (SE)	2.6 (0.01)	3.4 (0.01)	1.5 (0.01)	2.6 (0.01)	2.6 (0.01)	2.7 (0.01)
(n)	1,450	615	308	527	306	1,144
WAZ, mean (SE)	−0.42 (0.05)	−0.35 (0.08)	−0.40 (0.04)	−0.51 (0.10)	−0.31 (0.07)	−0.53 (0.05)
<−2, % (SE)	7.4 (0.01)	8.7 (0.01)	6.9 (0.02)	6.2 (0.02)	4.4 (0.01)	10.3 (0.02)
<−3, % (SE)	2.2 (0.01)	3.3 (0.02)	1.4 (0.01)	1.3 (0.01)	2.7 (0.02)	1.6 (0.00)

*Note*. HAZ: height‐for‐age z‐score; WHZ: weight‐for‐height z‐score; WAZ: weight‐for‐age z‐score.

a A total of 26 oedematous children was excluded out of 1,506 surveyed in National Nutrition Survey 2015. Out of the remaining 1,480 children, missing values or outlying values outside biologically reasonable ranges were identified as follows; HAZ (missing [*n* = 25]: outliers, HAZ < −6 or HAZ > 6 [*n* = 41]), WAZ (missing [*n* = 15]: outliers, WAZ < −6 or WAZ > 6 [*n* = 15]) and WHZ (missing [*n* = 35]: outliers, WHZ < −5 or WHZ > 5 [*n* = 12]).

b Sampling design of the original survey was taken into account in all analyses by using “svy” commands to estimate summary statistics at the population level.

**Figure 1:**
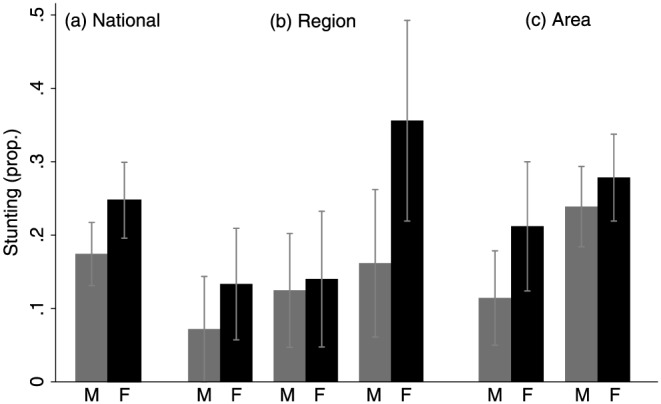
Prevalence of stunting in Bhutanese children aged 0 to 59 months by region, area and sex, from the National Nutrition Survey (NNS) 2015 in Bhutan (*n* = 1,414). M: male; F: female

The prevalence of stunting (<−2 HAZ) was 21.2%: higher in females (24.8%) than males (17.4%), increasing with age (from 5.3% at 0–5 months to 25.0% at 24–59 months), higher in the eastern (28.8%) than western (16.3%) or central (18.7%) regions, higher in rural (26.0%) than urban (16.2%) areas (Table 2, Figure 1, and Table S1) and decreasing with maternal education (from ~28.1% and 28.4% among children of mothers with no or only informal education to 21.2%, 12.9% and 0.4% among children whose mothers completed primary, high school and college education, respectively; Table S2). The prevalence of stunting also decreased with household wealth, from 34.8% in the lowest to 5.7% in the highest quintile. The prevalence of children with <−1 HAZ and <−3 HAZ was 48.0% and 6.2%, respectively, with each level similarly distributed by region and area as seen with stunting as classically defined (<−2 HAZ; Table S1).

The national prevalence of underweight (<−2 WAZ) was 7.4%, comparable by sex (5.5% vs 9.1% in males and females, respectively; Figure S3) and region (range: 6.2% to 8.7%), lower in urban (4.4%) than rural (10.3%) areas (Table 2), decreasing with maternal education (9.2% with none to 0.4% for college graduates), and similar for the all wealth quintiles, except that was greatest in the lowest wealth quintile (12.2%; Table S2).

### 3.4 Risk factors for wasting and overweight

None of the variables examined were significantly associated with continuous WHZ, wasting or overweight in multivariable models (data not shown).

### 3.5 Risk factors for stunting

In univariate regression analysis, HAZ was positively associated with wealth, improved housing (i.e., cement walls), water treatment, acceptable FCS and higher maternal education, and negatively associated with rural residence, lack of land ownership, lack of livestock ownership, receiving benefits from government programs and child age (*P* < 0.10 for all; Table 3). In multivariable regression analysis, only child age remained significantly negatively associated with HAZ (β = **−**0.03Z at 6–23 months [*P* = 0.07]; β = **−**1.69Z at 24–59 months [*P* = 0.01]; referent [0–6 months]).

**Table 3:** Predictors of height‐for‐age z‐score in the National Nutrition Survey 2015 (*n* = 1,414)

	Univariate			Multivariable		
	β	95% CI	*P*	β	95% CI	*P*
*Household*						
Region (Ref: West)						
Central	0.09	[−1.15, 1.33]	0.79			
East	−0.34	[−1.73, 1.05]	0.40			
Area (Ref: Urban)						
Rural	−0.48	[−1.02, 0.07]	0.06	−0.11	[−0.94, 0.73]	0.64
Wealth index quintiles (Ref: Lowest)^a^						
Low	0.36	[−0.63, 1.35]	0.26	0.33	[−0.49, 1.14]	0.23
Medium	0.42	[−0.35, 1.18]	0.15	0.43	[−0.36, 1.21]	0.15
High	0.80	[−0.24, 1.85]	0.08	0.70	[−0.38, 1.77]	0.11
Highest	1.02	[0.20, 1.84]	0.03	0.72	[−0.64, 2.08]	0.15
Wall materials (Ref: Mud)						
Cement	0.47	[−0.16.1.10]	0.08	−0.07	[−0.74, 0.60]	0.69
Owns land	−0.44	[−1.07, 0.18]	0.09	−0.03	[−0.59, 0.53]	0.82
Owns any livestock	−0.48	[−0.96, −0.01]	0.05	0.24	[−0.53, 1.00]	0.32
Improved water source^b^	−0.08	[−1.22, 1.06]	0.79			
Stored water	−0.19	[−0.74, 0.36]	0.28			
Water treatment^c^	0.67	[0.17, 1.18]	0.03	0.34	[−0.30, 0.96]	0.15
Improved sanitation	0.08	[−0.67, 0.82]	0.71	−0.26	[−0.98, 0.47]	0.27
Food insecure^d^	−0.28	[−1.33, 0.76]	0.36			
Food consumption score (Ref: borderline/poor)^e^						
Acceptable	0.85	[0.05, 1.65]	0.05	0.57	[−0.36, 1.50]	0.12
Benefited from government programmes	−0.35	[−0.80, 0.11]	0.08	−0.03	[−0.40, 0.34]	0.77
Household size (Ref: ≥6)						
<6	−0.34	[−0.84, 0.16]	0.10			
*Maternal*						
Education						
College	0.61	[−1.05, 2.27]	0.25	0.52	[−1.11, 2.14]	0.31
High school (Ref)	0.00	—	—	0.00	—	—
Primary	−0.50	[−1.47, 0.47]	0.16	−0.23	[−0.90, 0.43]	0.27
None	−0.44	[−1.20, 0.32]	0.13	−0.20	[−0.97, 0.57]	0.38
Informal	−0.65	[−1.42, 0.13]	0.07	−0.30	[−1.04, 0.43]	0.22
Age (Ref: <30 years)						
30–40 years	0.03	[−0.67, 0.73]	0.89			
≥41 years	−0.11	[−0.66, 0.45]	0.49			
*Child*						
Sex (Ref: Male)						
Female	−0.02	[−1.00, 0.96]	0.94	0.15	[−0.76, 1.07]	0.54
Age (Ref: 0–5 months)						
6–23 months	−0.98	[−2.16, 0.19]	0.07	−0.90	[−1.99, 0.18]	0.07
24–59 months	−1.80	[−3.05, −0.55]	0.03	−1.71	[−2.93, −0.49]	0.03

a Household asset score generated with principal component analysis as was done in the original analysis of the National Nutrition Survey data (Nutrition Program, Department of Public Health, Ministry of Health, 2016) but restricted to households with a child <5 years old who participated in the anthropometry assessment. The included variables were type of water source, shared sanitation facilities, toilet facilities, floor materials, roof materials, wall materials, cooking fuel, ownership of land, ownership of livestock, land of orchard, dry land, wet land, number of rooms, ownership of livestock, type of livestock (cattle/buffalo/yaks, pigs, horses, goats/sheep, poultry), sofa set, electric iron, bukhari, rice cooker, curry cooker, refrigerator, modern stove, water boiler, microwave oven, bicycle, tractor, power tiller, jewellery, motorbike/scooter, sechu gho/kira, family car, other vehicle, washing machine, sewing machine, television, VCR/VCD/DVD, grinding machine, wrist watch and weaving tool.

b Bhutan‐specific definition of household access to an improved water source is piped water into the household only.

c In the National Nutrition Survey 2015 survey, mothers were asked if she adds anything to the water to make it safer to drink.

d Food insecurity was one or more affirmative answers to the series of food security questions, represented as a composite variable of food insecurity that household experienced any out of four cases in the last month: (a) worry about not enough foods, (b) eat only rice/kharang/flour, (c) eat a smaller amount/skip meals at any meal time, and (d) eat fewer meals in a day.

e Food consumption score was based on household dietary diversity, food frequency, and relative nutritional importance of different food groups in the past 7 days (World Food Programme, 2008).

In univariate logistic analysis, the odds of stunting were higher, although not statistically significant, in rural compared with urban areas (OR = 1.82, 95% CI [0.85, 3.93], *P* = 0.08; Table 4). Risk was lower in wealthier households, evident by a lower OR if homes were in the highest versus lowest fifth of the wealth index distribution (OR = 0.11, 95% CI [0.02, 0.85], *P* = 0.04). The risk of stunting was lower in households practicing water treatment versus those who did not sanitizing water (OR = 0.43, 95% CI [0.15, 1.28], *P* = 0.08). Risk of childhood stunting was also lower among households reporting an acceptable FCS, compared with those reporting borderline or poor intakes (OR = 0.26, 95% CI [0.07, 0.90], *P* = 0.04). Expectedly, the odds of stunting increased with child age, reflected by an OR = 3.59 (95% CI [0.72, 18.0], *P* = 0.08) at 6–23 months and 5.92 (95% CI [1.36, 25.8], *P* = 0.04) at 24–59 months, relative to 0–5 months. Compared with children born to mothers with a high school education, selected as the referent, the odds of stunting increased in a dose–response manner among children whose mothers had less education (OR = 2.65, 95% CI [0.88, 7.99], *P* = 0.06 if mothers had none) and decreased for children of college educated mothers (OR = 0.02, 95% CI [0.00, 0.48], *P* = 0.03).

**Table 4:** Predictors of stunting (<−2 HAZ) in the National Nutrition Survey 2015 (*n* = 1,414)

	Univariate			Multivariable		
	OR	95% CI	*P*	OR	95% CI	*P*
Household level						
Region (Ref: West)	1.00					
Central	1.18	[0.24, 5.84]	0.70			
East	2.08	[0.61, 7.03]	0.12			
Area (Ref: Urban)	1.00			1.00		
Rural	1.82	[0.85, 3.93]	0.08	0.90	[0.29, 2.95]	0.82
Wealth index quintiles (Ref: Lowest)^a^	1.00			1.00		
Low	0.51	[0.11, 2.38]	0.20	0.53	[0.12, 2.36]	0.21
Medium	0.51	[0.14, 1.87]	0.16	0.67	[0.17, 2.63]	0.34
High	0.44	[0.11, 1.70]	0.12	0.63	[0.09, 4.26]	0.41
Highest	0.11	[0.02, 0.85]	0.04	0.19	[0.01, 3.55]	0.14
Wall materials (Ref: Mud)	1.00					
Cement	0.54	[0.22, 1.34]	0.10			
Owns land (Ref: No)	1.00					
Yes	1.22	[0.47, 3.20]	0.46			
Owns any livestock (Ref: No)	1.00					
Yes	1.52	[0.71, 3.26]	0.14			
Improved water source (Ref: No)^b^	1.00					
Yes	0.70	[0.17, 2.80]	0.38			
Stored water (Ref: No)	1.00					
Yes	1.40	[0.39, 4.96]	0.38			
Water treatment (Ref: No)^c^	1.00			1.00		
Yes	0.43	[0.15, 1.28]	0.08	0.74	[0.26, 2.13]	0.35
Improved sanitation (Ref: No)	1.00			1.00		
Yes	0.75	[0.30, 1.87]	0.30	1.20	[0.36, 4.05]	0.58
Food insecure (Ref: No)^d^	1.00					
Yes	2.45	[0.66, 9.07]	0.10			
Food consumption score (Ref: borderline/poor)^e^	1.00			1.00		
Acceptable	0.26	[0.07, 0.90]	0.04	0.31	[0.08, 1.28]	0.07
Benefited from government programmes (Ref: No)	1.00					
Yes	1.19	[0.42, 3.38]	0.55			
Household size (Ref: ≥6)	1.00					
<6	1.03	[0.43, 2.49]	0.90			
Maternal level						
Education						
College	0.02	[0.00, 0.48]	0.03	0.07	[0.00, 1.20]	0.06
High school (Ref)	1.00	—	—	1.00	—	—
Primary	1.82	[0.34, 9.84]	0.27	1.29	[0.30, 5.60]	0.54
None	2.65	[0.88, 7.99]	0.06	1.78	[0.51, 6.25]	0.19
Informal	2.69	[0.58, 12.4]	0.11	1.75	[0.37, 8.22]	0.26
Age (Ref: <30 years)	1.00					
30–40 years	0.75	[0.30, 1.84]	0.30			
≥41 years	0.80	[0.14, 4.65]	0.64			
Child level						
Sex (Ref: Male)	1.00			1.00		
Female	1.56	[0.70, 3.49]	0.14	1.26	[0.58, 2.73]	0.32
Age (Ref: 0–5 months)	1.00			1.00		
6–23 months	3.59	[0.72, 18.0]	0.08	3.06	[0.63, 14.8]	0.09
24–59 months	5.92	[1.36, 25.8]	0.04	5.07	[1.16, 22.2]	0.04

a Household asset score generated with principal component analysis as was done in the original analysis of the National Nutrition Survey data (Nutrition Program, Department of Public Health, Ministry of Health, 2016) but restricted to households with a child <5 years old who participated in the anthropometry assessment. The included variables were type of water source, shared sanitation facilities, toilet facilities, floor materials, roof materials, wall materials, cooking fuel, ownership of land, ownership of livestock, land of orchard, dry land, wet land, number of rooms, ownership of livestock, type of livestock (cattle/buffalo/yaks, pigs, horses, goats/sheep, poultry), sofa set, electric iron, bukhari, rice cooker, curry cooker, refrigerator, modern stove, water boiler, microwave oven, bicycle, tractor, power tiller, jewellery, motorbike/scooter, sechu gho/kira, family car, other vehicle, washing machine, sewing machine, television, VCR/VCD/DVD, grinding machine, wrist watch and weaving tool.

b Bhutan‐specific definition of household access to an improved water source is piped water into the household only.

c In the National Nutrition Survey 2015 survey, mothers were asked if she adds anything to the water to make it safer to drink.

d Food insecurity was one or more affirmative answers to the series of food security questions, represented as a composite variable of food insecurity that household experienced any out of four cases in the last month: (a) worry about not enough foods, (b) eat only rice/kharang/flour, (c) eat a smaller amount/skip meals at any meal time and (d) eat fewer meals in a day.

e Food consumption score was based on household dietary diversity, food frequency, and relative nutritional importance of different food groups in the past 7 days (World Food Programme, 2008).

On multivariable adjustment, only older child age remained a risk factor for stunting, reflected by an OR = 3.06 (95% CI [0.63, 14.8], *P* = 0.09) and OR = 5.07 (95% CI [1.16, 22.2], *P* = 0.04) for ages of 6–23 and 24–59 months, respectively, versus the 0–5 months of age group. While odds ratios were attenuated, a dose–response relationship between risk of stunting and maternal education remained evident in point estimates. In subgroup multivariable analysis by age group (6–23 months and 24–59 months), none of the risk factors remained significant for stunting, except for a dose–response relationship with maternal education among children 6–23 months of age was observed (Tables S3 and S4).

Similar to classically defined stunting (<−2 HAZ), variables associated with a higher odds of <−1 HAZ included rural residence, livestock ownership, benefits from government programs and child age in univariate analyses, and lower odds were observed with wealth, improved housing, practice of water treatment, and acceptable FCS at household level (*P* < 0.10 for all; Table 5). In a multivariable analysis, child age remained the sole risk factor of <−1 HAZ. The risk was higher at 6–23 months of age (OR = 4.50, 95% CI [1.38, 14.7], *P* = 0.03) and at 24–59 months of age (OR = 11.4, 95% CI [2.37, 55.1], *P* = 0.02), compared with 0–6 months of age. An observed dose–response, inverse relationship between the odds of <−1 HAZ and wealth was not significant.

**Table 5:** Predictors of <−1 HAZ in the National Nutrition Survey 2015 (*n* = 1,414)

	Univariate			Multivariable		
	OR	95% CI	*P*	OR	95% CI	*P*
Household level						
Region (Ref: West)	1.00					
Central	0.99	[0.17, 5.89]	0.98			
East	1.43	[0.21, 9.72]	0.51			
Area (Ref: Urban)	1.00			1.00		
Rural	1.78	[0.93, 3.40]	0.06	1.11	[0.36, 3.42]	0.72
Wealth index quintiles (Ref: Lowest)^a^	1.00			1.00		
Low	0.66	[0.25, 1.74]	0.20	0.66	[0.26, 1.67]	0.20
Medium	0.60	[0.23, 1.59]	0.15	0.61	[0.21, 1.79]	0.19
High	0.41	[0.13, 1.34]	0.08	0.44	[0.08, 2.39]	0.17
Highest	0.25	[0.06, 1.12]	0.06	0.25	[0.02, 3.19]	0.14
Wall materials (Ref: Mud)	1.00			1.00		
Cement	0.56	[0.27, 1.16]	0.08	1.12	[0.34, 3.74]	0.72
Owns land (Ref: No)	1.00					
Yes	1.61	[0.76, 3.43]	0.11			
Owns any livestock (Ref: No)	1.00			1.00		
Yes	1.85	[0.98, 3.49]	0.05	0.87	[0.20, 3.77]	0.73
Improved water source (Ref: No)^b^	1.00					
Yes	0.80	[0.26, 2.47]	0.48			
Stored water (Ref: No)	1.00					
Yes	1.31	[0.49, 3.54]	0.36			
Water treatment (Ref: No)^c^	1.00			1.00		
Yes	0.43	[0.15, 1.27]	0.08	0.68	[0.21, 2.14]	0.29
Improved sanitation (Ref: No)	1.00			1.00		
Yes	0.71	[0.38, 1.35]	0.15	1.01	[0.50, 2.04]	0.95
Food insecure (Ref: No)^d^	1.00					
Yes	1.61	[0.12, 22.1]	0.52			
Food consumption score (Ref: borderline/poor)^e^	1.00			1.00		
Acceptable	0.30	[0.10, 0.91]	0.04	0.39	[0.13, 1.18]	0.07
Benefited from government programmes (Ref: No)	1.00			1.00		
Yes	1.66	[0.84, 3.27]	0.09	1.15	[0.51, 2.62]	0.54
Household size (Ref: ≥6)	1.00					
<6	1.50	[0.84, 2.71]	0.10			
Maternal level						
Education						
College	0.35	[0.01, 12.4]	0.33			
High school (Ref)	1.00	—	—			
Primary	2.06	[0.21, 20.1]	0.31			
None	1.83	[0.70, 4.78]	0.11			
Informal	2.04	[0.58, 7.17]	0.14			
Age (Ref: <30 years)	1.00					
30–40 years	1.00	[0.45, 2.22]	0.99			
≥41 years	1.48	[0.65, 3.37]	0.18			
Child level						
Sex (Ref: Male)	1.00			1.00		
Female	1.29	[0.58, 2.90]	0.31	1.16	[0.50, 2.66]	0.53
Age (Ref: 0–5 months)	1.00			1.00		
6–23 months	4.64	[1.41, 15.2]	0.03	4.39	[1.30, 14.8]	0.03
24–59 months	10.8	[2.54, 45.6]	0.02	11.2	[2.27, 54.8]	0.02

a Household asset score generated with principal component analysis as was done in the original analysis of the National Nutrition Survey data (Nutrition Program, Department of Public Health, Ministry of Health, 2016) but restricted to households with a child <5 years old who participated in the anthropometry assessment. The included variables were type of water source, shared sanitation facilities, toilet facilities, floor materials, roof materials, wall materials, cooking fuel, ownership of land, ownership of livestock, land of orchard, dry land, wet land, number of rooms, ownership of livestock, type of livestock (cattle/buffalo/yaks, pigs, horses, goats/sheep, poultry), sofa set, electric iron, bukhari, rice cooker, curry cooker, refrigerator, modern stove, water boiler, microwave oven, bicycle, tractor, power tiller, jewellery, motorbike/scooter, sechu gho/kira, family car, other vehicle, washing machine, sewing machine, television, VCR/VCD/DVD, grinding machine, wrist watch and weaving tool.

b Bhutan‐specific definition of household access to an improved water source is piped water into the household only.

c In the National Nutrition Survey 2015 survey, mothers were asked if she adds anything to the water to make it safer to drink.

d Food insecurity was one or more affirmative answers to the series of food security questions, represented as a composite variable of food insecurity that household experienced any out of four cases in the last month: (a) worry about not enough foods, (b) eat only rice/kharang/flour, (c) eat a smaller amount/skip meals at any meal time and (d) eat fewer meals in a day.

e Food consumption score was based on household dietary diversity, food frequency and relative nutritional importance of different food groups in the past 7 days (World Food Programme, 2008).

Rural versus urban residence was associated with a higher odds of <−3 HAZ in univariate analysis, and medium wealth intervals were associated with lower odds than the lowest group (*P* < 0.10; Table S5 **)**. None of assessed variables were associated with risk of <−3 HAZ on multivariate adjustment.

### 3.6 Risk factors for underweight

Similar to HAZ, in univariate analysis, indicators of socioeconomic status, improved housing, water treatment, and acceptable food consumption were positively associated, and child age was inversely associated, with WAZ (*P* < 0.10; Table S6). In multivariable models, however, only child age in months remained a significant risk factor, reflected by β = −0.50 (95% CI [−1.60, 0.61], *P* = 0.17) at 6–23 months and β = −1.23 (95% CI [1.36, 25.8], *P* = 0.04) at 24–59 months, relative to 0–5 months. With respect to underweight (<−2 WAZ), rural children were at greater risk than urban, and a dose–response association was evident by levels of formal education of mothers, although effect estimates were not statistically significant (Table S7).

### 3.7 Time trend of nutritional status

**Figure 2:**
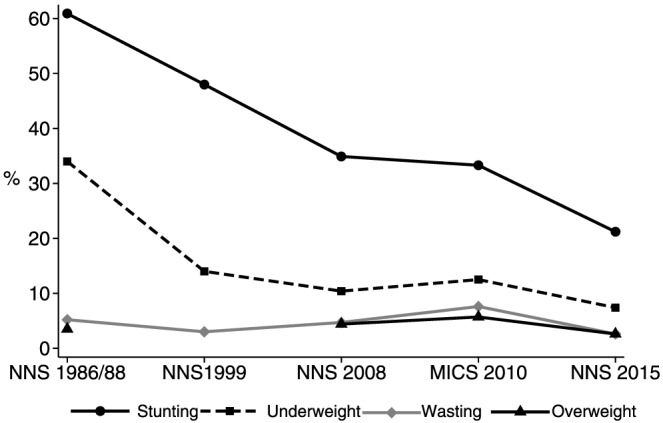
Prevalence of stunting, underweight, wasting and overweight among Bhutanese children aged 0 to 59 months from National Nutrition Survey (NNS) 1986/1988, NNS 1999, NNS 2008, Multiple Indicator Cluster Survey (MICS) 2010, and NNS 2015. Overweight data were not available at NNS 1999. The nutrition indicators in the MICS 2010 were estimated using dataset available at MICS site (http://mics.unicef.org/). Other nutrition indicators were excerpted from Zangmo et al. (2012), NNS 1968/1988 (Directorate of Health Services, 1989) and NNS 1999 (Namgyal & Yoezer, 1999). The prevalence of nutrition indicators in 2015 was estimated, excluding 26 oedematous children out of 1,506 children recruited in the NNS 2015 report

The prevalence of stunting decreased markedly from 60.9% in the NNS conducted from 1986 to 1988 to 21.2% in the NNS of 2015 (Figure 2), representing an average 1.43 percentage point (pp) reduction per year. The reduction in underweight was also remarkable during the same period, declining from 34.0% to 7.4%. Neither wasting nor overweight showed a significant time trend, with the prevalence for both remaining between 3% and 8%.

## 4 DISCUSSION

Findings from the 2015 NNS provide a comprehensive overview of the nutritional status of Bhutanese preschool children by geographic area, urban–rural residence and other common risk factors. The dominant national pattern is one of persistent stunting, affecting one fifth of children, amidst a documented steady decrease in the prevalence of stunting since 1986. Notwithstanding a steady decline, stunting continues to be a public health and development problem in Bhutan with a burden consistent with other low‐ and middle‐income countries in the Asian region. For example, a prevalence of 21% is similar in magnitude to rates in middle‐income Asian countries, including Vietnam (23%; General Statistics Office of Vietnam, 2011) and Tajikistan (27%; UNICEF, 2016), while remaining moderate for South Asia, following Sri Lanka (15%) and Maldives (17%) but lower than an overall prevalence of stunting of ~37% among young children in neighbouring Bangladesh, India and Nepal (UNICEF, 2016).

On the other hand, the weight‐for‐height *z*‐score distribution of preschool children is largely superimposed on the WHO reference curve (WHO Multicentre Growth Reference Study Group, 2006), yielding prevalence estimates of ~2.6% below and above the conventional 2 *z*‐score cut‐offs for wasting and overweight, respectively, as would be approximately expected under the curve. We did not find significant risk factors for neither wasting nor overweight in our analysis. The observed weight‐for‐height distribution and low prevalence of wasting is also consistent with a few national nutrition and health indicators reflecting low rates of household food insecurity (2%), high rates of access to improved water sources (98.3%) and sanitation facilities (70.6%), and high rates of vaccination (87%) and deworming coverage (89%; Nutrition Program, Department of Public Health, Ministry of Health, 2016). The observed, dissociated pattern whereby a substantial proportion of children are stunted in the presence of a normal WHZ distribution suggests that a significant number of children with a seemingly “healthy” weight may nonetheless lack nutrients or face nonnutritional stresses that constrain linear growth.

Our study showed an expected higher risk of stunting among older than younger children, consistent with a commonly observed deceleration in linear growth of children through 2 years of age, followed by insufficient recovery thereafter in most impoverished settings (de Onis & Branca, 2016). Our age‐stratified analysis, however, did not find any stratum‐specific risk factors, likely due to small sample size leading to reduced study power to detect differences.

Although not significant, an inverse trend in stunting may have existed with maternal education, as seen elsewhere in the region (Aguayo, Badgaiyan, & Dzed, 2017; Dorsey et al., 2017), possibly related to factors other than feeding practices, which recently were not found to be associated with early childhood stunting in Bhutan (Campbell, Aguayo, et al., 2018).

Analyses of the MICS conducted in 2010 in Bhutan reported risk factors for early childhood undernutrition, focusing on children under 24 months of age (Aguayo et al., 2015, 2017). Identified determinants of stunting included child age, residency in the eastern or western region (relative to Central), low socioeconomic status and suboptimal complementary feeding practices among children under one year of age (Aguayo et al., 2015; Zangmo et al., 2012), and determinants of wasting were child age, residency in the western region and inappropriate complementary feeding practices (Aguayo et al., 2017). Differences in identifying risk factors between the 2010 and 2015 surveys might have arisen from the latter study's wider age range, smaller sample size or different design but may also be reflecting, in part, a process of continued socioeconomic development in rural areas in the interim years (Centre For Bhutan Studies & Gross National Happiness Research, 2018; National Statistics Bureau Royal Government of Bhutan & World Bank, 2017).

Additional factors related to stunting to investigate should include environmental enteric dysfunction (Campbell, Schulze, et al., 2017), potential roles of micronutrient deficiencies (e.g., zinc) and other possible exposures such as aflatoxin, as recently reported to be endemic in neighbouring countries of Nepal and Bangladesh (Groopman et al., 2014).

Dzed and Wangmo have suggested through their ecological analysis that recent national efforts to modernize, increase trade, eradicate extreme poverty and implement expanded health and nutrition programs may be contributing to the reductions in childhood stunting seen in Bhutan (Dzed & Wangmo, 2016), similar to posited effects of poverty alleviation on reductions in stunting in Nepal and Vietnam (Headey & Hoddinott, 2015). For example, based on findings from the Nepal Demographic and Health Survey 2011, increased household wealth, improved food security, optimal breastfeeding practices and residence in the hill versus mountain agroecological zones were associated with a lower risk of stunting in preschool‐aged children (Tiwari, Ausman, & Agho, 2014). In Vietnam, MICS 2000 and 2011 findings have identified rising maternal education as a critical explanatory factor for an observed reduction in stunting (Kien et al., 2016).

Previous trend analyses for Bhutan have reported an average decline in stunting in preschool‐aged children of ~1.3 pp per year between 1986/1988 to 2008 (Zangmo et al., 2012). Our analysis has added two further time points, in 2010 and 2015, to show an average reduction of 1.96 pp per year in the prevalence of stunting in the subsequent seven years (2008–2015), yielding an average reduction of 1.43 pp per year since 1986/1988. This average annual rate of reduction in Bhutan exceeds estimates of 1.01 pp per year in Nepal (i.e., from 50.5% in 2001 to 37.4% in 2014; Ministry of Health [Nepal], New ERA, & ORC Macro, 2002; Nepal Central Bureau of Statistics, 2015) and 1.25 pp per year in Vietnam (i.e., from 36.4% in 2000 to 22.7% in 2011; General Statistics Office of Vietnam, 2011; Vietnam Statistical Publishing House, 2000).

Prevalence rates of wasting (2.6%) and underweight (7.4%) estimated in the current analysis are slightly lower than rates of 4.3% and 9.0%, respectively, reported earlier from the Bhutan NNS 2015 (Nutrition Program, Department of Public Health, Ministry of Health, 2016). These differences are explained by our excluding from anthropometric status estimates children who, during the survey, were clinically assessed to have pedal oedema (*n* = 26), a condition that could distort estimates of weight‐for‐height and weight‐for‐age. Oedematous children were comparable with noncases with respect to height‐for‐age, dietary intake and morbidity except for a higher percentage with reported diarrhoea in the previous two weeks (17.4% vs. 6.9%, *P* = 0.05;Table S9). Combining these cases with children classified as wasted and underweight yields the same rates reported earlier (Nutrition Program, Department of Public Health, Ministry of Health, 2016).

Our study has a few limitations. First, a complex sampling design and relatively small sample size for a national survey may have increased variability in outcome assessments, including HAZ, compared with previous surveys, with consequent reduced power to detect associations. Secondly, variables with many missing observations were excluded in the association analysis to avoid sample size loss. For example, less data was available for antenatal and postnatal care, maternal hygienic and sanitation (hand washing practices and safe disposal of child stools) and child morbidity (*n* < 1,100), which could have been predictors of stunting.

In conclusion, a moderate level of stunting persists in preschool‐aged children in Bhutan despite low risk of wasting, suggesting a need to further understand risk factors and potential longer term nutritional or nonnutritional interventions that may help lower the burden of depressed linear growth in the country.

## CONFLICTS OF INTEREST

The authors declare that they have no conflicts of interest. The contents expressed in the article are those of the authors and do not necessarily reflect the policies or views of the organizations that they are affiliated with.

## CONTRIBUTIONS

The authors' responsibilities were as follows—YK, RC, VA, and KW designed the current study; VA, LD, JW, SG, and NH led survey design, implementation, and analysis for the National Nutrition Survey 2015; YK and RC analysed data, and YK wrote the manuscript; RC, KW, VA, LD, VJ, JW, SG, and NH reviewed the manuscript and substantially contributed to interpretation of the results; KW had primary responsibility for final content. All authors read and approved the final manuscript.

## Supporting information

Figure S1. Prevalence of wasting in Bhutanese children aged 0 to 59 months by region, area and sex, from the National Nutrition Survey (NNS) 2015 (*n* = 1,433). M: male; F: female

Figure S2. Prevalence of overweight in Bhutanese children aged 0 to 59 months by region, area and sex, from the National Nutrition Survey (NNS) 2015 (*n* = 1,433). M: male; F: female

Figure S3. Prevalence of underweight in Bhutanese children aged 0 to 59 months by region, area and sex, from the National Nutrition Survey (NNS) 2015 (*n* = 1,450). M: male; F: female

Table S1. Nutritional status among children aged 0 to 59 months by age, region and area in the National Nutrition Survey (NNS) 2015

Table S2. Nutritional status among children aged 0 to 59 months by wealth index and maternal education from the National Nutrition Survey (NNS) 2015

Table S3. Predictors of stunting (<−2 HAZ) in the National Nutrition Survey (NNS) 2015 among 6–23 months (*n* = 436)

Table S4. Predictors of stunting (<−2 HAZ) in the National Nutrition Survey (NNS) 2015 among 24–59 months (*n* = 864)

Table S5. Predictors of <−3 HAZ in the National Nutrition Survey (NNS) 2015 (*n* = 1,414)

Table S6. Predictors of WAZ in the National Nutrition Survey (NNS) 2015 (*n* = 1,450)

Table S7. Predictors of underweight in the National Nutrition Survey (NNS) 2015 (n = 1,450)

Table S8. Child nutritional status, morbidity, and dietary practices in children with and without oedema in National Nutrition Survey 2015^1^
