# Supplementary material for: Nutritional status and risk factors for stunting in preschool children in Bhutan
Source: Matern Child Nutr. 2018 Nov 9;14(Suppl 4):e12653. doi: 10.1111/mcn.12653 (PMC6587444; doi:10.1111/mcn.12653)
Supplement: Supplementary file 2 — Figure S2. Prevalence of overweight in Bhutanese children aged 0 to 59 months by region, area and sex, from the National Nutrition Survey (NNS) 2015 (n = 1,433). M: male; F: female [file MCN-14-e12653-s002.docx]

Supplemental Figure 2. Prevalence of overweight in Bhutanese children aged 0 to 59 months by region, area and sex, from the National Nutrition Survey (NNS) 2015 (n=1,433). M: male; F: female
