# Supplementary material for: Nutritional status and risk factors for stunting in preschool children in Bhutan
Source: Matern Child Nutr. 2018 Nov 9;14(Suppl 4):e12653. doi: 10.1111/mcn.12653 (PMC6587444; doi:10.1111/mcn.12653)
Supplement: Supplementary file 4 — Table S1. Nutritional status among children aged 0 to 59 months by age, region and area in the National Nutrition Survey (NNS) 2015 [file MCN-14-e12653-s004.docx]

**Supplemental Table 1. Nutritional status among children aged 0 to 59 months by age, region and area in the National Nutrition Survey (NNS) 2015**

|  | National | Region | | | Area | |
| --- | --- | --- | --- | --- | --- | --- |
|  |  | West | Central | East | Urban | Rural |
| n | 1,414 | 600 | 302 | 512 | 301 | 1,138 |
| Height-for-age z-score (HAZ), Mean (SE) |  |  |  |  |  |  |
| 0 to 5 mo | 0.61 (0.31) | 0.36 (0.37) | 0.85 (0.37) | 0.82 (0.62) | 1.15 (0.40) | -0.11 (0.29) |
| 6 to 23 mo | -0.37 (0.19) | -0.37 (0.39) | 0.44 (0.15) | -0.99 (0.19) | -0.34 (0.20) | -0.39 (0.25) |
| 24 to 59 mo | -1.18 (0.09) | -1.05 (0.17) | -1.19 (0.09) | -1.33 (0.18) | -0.89 (0.13) | -1.49 (0.09) |
| <-1 HAZ, % (SE) |  |  |  |  |  |  |
| 0 to 5 mo | 11.1 (0.05) | 14.8 (0.07) | 9.7 (0.09) | 7.3 (0.03) | 0.1 (0.00) | 26.2 (0.07) |
| 6 to 23 mo | 36.7 (0.07) | 39.1 (0.14) | 23.3 (0.05) | 43.5 (0.07) | 35.5 (0.08) | 37.7 (0.07) |
| 24 to 59 mo | 57.3 (0.03) | 52.1 (0.08) | 56.3 (0.03) | 63.9 (0.05) | 48.0 (0.04) | 66.9 (0.03) |
| <-2 HAZ, % (SE) |  |  |  |  |  |  |
| 0 to 5 mo | 5.3 (0.02) | 7.7 (0.02) | 0.0 | 4.6 (0.03) | 0.0 | 12.6 (0.04) |
| 6 to 23 mo | 16.8 (0.03) | 17.6 (0.06) | 5.8 (0.01) | 24.1 (0.06) | 11.9 (0.04) | 20.6 (0.04) |
| 24 to 59 mo | 25.0 (0.02) | 16.8 (0.02) | 25.4 (0.06) | 33.7 (0.04) | 19.9 (0.03) | 30.1 (0.03) |
| <-3 HAZ, % (SE) |  |  |  |  |  |  |
| 0 to 5 mo | 3.7 (0.02) | 4.3 (0.04) | 0.0 | 4.4 (0.03) | 0.0 | 8.8 (0.04) |
| 6 to 23 mo | 5.1 (0.01) | 3.8 (0.01) | 1.8 (0.01) | 9.5 (0.03) | 4.0 (0.02) | 5.9 (0.02) |
| 24 to 59 mo | 7.0 (0.02) | 5.3 (0.03) | 9.5 (0.03) | 7.1 (0.03) | 3.7 (0.02) | 10.5 (0.02) |
|  |  |  |  |  |  |  |
| N | 1,433 | 609 | 304 | 520 | 299 | 1,134 |
| Weight-for-height z-score (WHZ), Mean (SE) |  |  |  |  |  |  |
| 0 to 5 mo | 0.26 (0.11) | 0.35 (0.15) | 0.12 (0.34) | 0.19 (0.15) | 0.11 (0.21) | 0.50 (0.16) |
| 6 to 23 mo | 0.25 (0.06) | 0.34 (0.06) | 0.01 (0.07) | 0.32 (0.18) | 0.41 (0.10) | 0.13 (0.10) |
| 24 to 59 mo | 0.01 (0.06) | -0.04 (0.10) | -0.05 (0.06) | 0.10 (0.11) | -0.04 (0.09) | 0.06 (0.06) |
| <-2 WHZ, % (SE) |  |  |  |  |  |  |
| 0 to 5 mo | 4.0 (0.03) | 1.9 (0.02) | 18.2 (0.15) | 1.4 (0.01) | 4.1 (0.04) | 3.8 (0.02) |
| 6 to 23 mo | 2.6 (0.01) | 1.6 (0.01) | 2.1 (0.01) | 4.7 (0.03) | 0 | 4.6 (0.02) |
| 24 to 59 mo | 2.3 (0.01) | 2.4 (0.01) | 2.5 (0.01) | 2.1 (0.02) | 2.4 (0.01) | 2.3 (0.01) |
| <-3 WHZ, % (SE) |  |  |  |  |  |  |
| 0 to 5 mo | 0.0 | 0.0 | 0.0 | 0.0 | 0.0 | 0.0 |
| 6 to 23 mo | 0.4 (0.00) | 0.8 (0.01) | 0.0 | 0.0 | 0.0 | 0.6 (0.01) |
| 24 to 59 mo | 0.5 (0.00) | 1.0 (0.00) | 0.3 (0.00) | 0.0 | 0.4 (0.00) | 0.5 (0.00) |
| >2 WHZ, % (SE) |  |  |  |  |  |  |
| 0 to 5 mo | 7.6 (0.03) | 11.2 (0.05) | 10.0 (0.09) | 2.6 (0.02) | 6.2 (0.05) | 9.9 (0.05) |
| 6 to 23 mo | 3.9 (0.01) | 5.0 (0.02) | 1.9 (0.02) | 3.8 (0.02) | 4.0 (0.02) | 3.9 (0.01) |
| 24 to 59 mo | 1.3 (0.01) | 1.2 (0.01) | 0.7 (0.01) | 2.0 (0.02) | 1.4 (0.01) | 1.2 (0.01) |
|  |  |  |  |  |  |  |
| N | 1,450 | 615 | 308 | 527 | 306 | 1,144 |
| Weight-for-age z-score (WAZ), Mean (SE) |  |  |  |  |  |  |
| 0 to 5 mo | 0.54 (0.27) | 0.71 (0.39) | 0.36 (0.65) | 0.42 (0.47) | 0.84 (0.34) | 0.16 (0.28) |
| 6 to 23 mo | 0.01 (0.08) | 0.06 (0.13) | 0.18 (0.09) | -0.20 (0.14) | 0.11 (0.12) | -0.08 (0.08) |
| 24 to 59 mo | -0.72 (0.04) | -0.72 (0.06) | -0.70 (0.06) | -0.75 (0.09) | -0.62 (0.09) | -0.84 (0.05) |
| <-2 WAZ, % (SE) |  |  |  |  |  |  |
| 0 to 5 mo | 4.7 (0.02) | 4.9 (0.02) | 0.0 | 6.2 (0.05) | 0.0 | 10.7 (0.05) |
| 6 to 23 mo | 5.7 (0.01) | 5.8 (0.03) | 5.3 (0.01) | 5.8 (0.02) | 3.7 (0.02) | 7.3 (0.02) |
| 24 to 59 mo | 8.5 (0.01) | 10.8 (0.02) | 8.0 (0.03) | 6.4 (0.02) | 5.2 (0.01) | 11.9 (0.02) |
| <-3 WAZ, % (SE) |  |  |  |  |  |  |
| 0 to 5 mo | 2.4 (0.02) | 0.2 (0.00) | 0.0 | 5.9 (0.05) | 0.0 | 5.5 (0.04) |
| 6 to 23 mo | 1.4 (0.01) | 2.8 (0.01) | 0.0 | 0.4 (0.00) | 2.0 (0.02) | 0.9 (0.01) |
| 24 to 59 mo | 2.5 (0.01) | 4.0 (0.03) | 2.1 (0.02) | 1.1 (0.01) | 3.3 (0.02) | 1.7 (0.01) |
