# Supplementary material for: Nutritional status and risk factors for stunting in preschool children in Bhutan
Source: Matern Child Nutr. 2018 Nov 9;14(Suppl 4):e12653. doi: 10.1111/mcn.12653 (PMC6587444; doi:10.1111/mcn.12653)
Supplement: Supplementary file 5 — Table S2. Nutritional status among children aged 0 to 59 months by wealth index and maternal education from the National Nutrition Survey (NNS) 2015 [file MCN-14-e12653-s005.docx]

**Supplemental Table 2. Nutritional status among children aged 0 to 59 months by wealth index and maternal education from the National Nutrition Survey (NNS) 2015**

|  | Stunting  % (SE) | Wasting  % (SE) | Underweight  % (SE) | Overweight  % (SE) |
| --- | --- | --- | --- | --- |
| **Wealth status** |  |  |  |  |
| *n* | 1,414 | 1,433 | 1,450 | 1,433 |
| Poorest | 34.8 (0.04) | 4.0 (0.01) | 12.2 (0.03) | 2.9 (0.01) |
| Poor | 21.6 (0.04) | 1.8 (0.01) | 6.9 (0.02) | 2.1 (0.02) |
| Medium | 21.5 (0.04) | 1.8 (0.01) | 6.1 (0.02) | 3.6 (0.02) |
| Wealthy | 19.0 (0.04) | 3.4 (0.02) | 5.9 (0.02) | 2.5 (0.01) |
| Wealthiest | 5.7 (0.02) | 1.7 (0.01) | 5.6 (0.03) | 1.7 (0.01) |
|  |  |  |  |  |
| ***Education*** |  |  |  |  |
| *n* | 1,347 | 1,365 | 1,380 | 1,365 |
| College | 0.4 (0.00) | 0.0 (0.00) | 0.4 (0.00) | 1.9 (0.02) |
| High school | 12.9 (0.02) | 1.4 (0.01) | 5.3 (0.01) | 4.2 (0.02) |
| Primary | 21.2 (0.05) | 1.8 (0.01) | 8.3 (0.02) | 0.8 (0.01) |
| None | 28.1 (0.03) | 4.3 (0.01) | 9.2 (0.02) | 2.4 (0.01) |
| Informal | 28.4 (0.05) | 2.3 (0.02) | 5.4 (0.02) | 3.0 (0.02) |
