# Supplementary material for: Nutritional status and risk factors for stunting in preschool children in Bhutan
Source: Matern Child Nutr. 2018 Nov 9;14(Suppl 4):e12653. doi: 10.1111/mcn.12653 (PMC6587444; doi:10.1111/mcn.12653)
Supplement: Supplementary file 8 — Table S5. Predictors of <−3 HAZ in the National Nutrition Survey (NNS) 2015 (n = 1,414) [file MCN-14-e12653-s008.docx]

**Supplemental Table 5. Predictors of <-3 HAZ in the National Nutrition Survey (NNS) 2015 (n=1,414)**

|  | Univariate | | | Multivariable | | |
| --- | --- | --- | --- | --- | --- | --- |
|  | OR | 95% CI | P | OR | 95% CI | P |
| ***Household level*** |  |  |  |  |  |  |
| Region (Ref: West) | 1.00 |  |  |  |  |  |
| Central | 1.49 | 0.13, 16.7 | 0.55 |  |  |  |
| East | 1.64 | 0.18, 14.9 | 0.44 |  |  |  |
| Area (Ref: Urban) | 1.00 |  |  | 1.00 |  |  |
| Rural | 2.72 | 0.69, 10.7 | 0.09 | 1.98 | 0.46, 8.61 | 0.18 |
| Wealth index quintiles (Ref: Lowest)^1^ | 1.00 |  |  | 1.00 |  |  |
| Low | 0.38 | 0.04, 4.01 | 0.22 | 0.37 | 0.03, 5.07 | 0.24 |
| Medium | 0.28 | 0.06, 1.44 | 0.08 | 0.39 | 0.04, 3.67 | 0.21 |
| High | 0.53 | 0.04, 6.85 | 0.40 | 0.70 | 0.04, 12.0 | 0.65 |
| Highest | 0.13 | 0.00, 4.09 | 0.13 | 0.19 | 0.00, 9.27 | 0.21 |
| Wall materials (Ref: Mud) | 1.00 |  |  |  |  |  |
| Cement | 0.58 | 0.16, 2.06 | 0.21 |  |  |  |
| Owns land (Ref: No) | 1.00 |  |  |  |  |  |
| Yes | 1.25 | 0.34, 4.60 | 0.54 |  |  |  |
| Owns any livestock (Ref: No) | 1.00 |  |  |  |  |  |
| Yes | 2.02 | 0.51, 8.03 | 0.16 |  |  |  |
| Improved water source (Ref: No)^2^ | 1.00 |  |  |  |  |  |
| Yes | 1.52 | 0.22, 10.5 | 0.45 |  |  |  |
| Stored water (Ref: No) | 1.00 |  |  |  |  |  |
| Yes | 0.95 | 0.24, 3.81 | 0.89 |  |  |  |
| Water treatment (Ref: No)^3^ | 1.00 |  |  |  |  |  |
| Yes | 0.49 | 0.10, 2.47 | 0.20 |  |  |  |
| Improved sanitation (Ref: No) | 1.00 |  |  | 1.00 |  |  |
| Yes | 1.21 | 0.30, 4.87 | 0.61 | 1.86 | 0.32, 10.8 | 0.27 |
| Food insecure (Ref: No)^4^ | 1.00 |  |  |  |  |  |
| Yes | 0.39 | 0.02, 7.84 | 0.31 |  |  |  |
| Food consumption score (Ref: Borderline/poor)^5^ | 1.00 |  |  |  |  |  |
| Acceptable | 0.57 | 0.09, 3.69 | 0.33 |  |  |  |
| Benefited from any govern­ment programmes (Ref: No) | 1.00 |  |  |  |  |  |
| Yes | 1.66 | 0.43, 6.37 | 0.25 |  |  |  |
| Household size (Ref: ≥6) | 1.00 |  |  |  |  |  |
| <6 | 1.80 | 0.41, 7.88 | 0.23 |  |  |  |
|  |  |  |  |  |  |  |
| ***Maternal level*** |  |  |  |  |  |  |
| Education |  |  |  |  |  |  |
| College | 0.09 | 0.00, 7.42 | 0.15 |  |  |  |
| High school (Ref) | 1.00 | - | - |  |  |  |
| Primary | 1.30 | 0.09, 19.8 | 0.72 |  |  |  |
| None | 1.89 | 0.38, 9.28 | 0.23 |  |  |  |
| Informal | 2.83 | 0.36, 22.5 | 0.16 |  |  |  |
| Age (Ref:<30 years) | 1.00 |  |  |  |  |  |
| 30-40 years | 1.54 | 0.36, 6.49 | 0.33 |  |  |  |
| ≥41 years | 0.40 | 0.02, 6.64 | 0.29 |  |  |  |
|  |  |  |  |  |  |  |
| ***Child level*** |  |  |  |  |  |  |
| Sex (Ref: Male) | 1.00 |  |  | 1.00 |  |  |
| Female | 1.62 | 0.42, 6.30 | 0.27 | 1.48 | 0.38, 5.72 | 0.34 |
| Age (Ref: 0-5 months) | 1.00 |  |  |  |  |  |
| 6-23 months | 1.39 | 0.12, 16.5 | 0.63 |  |  |  |
| 24-59 months | 1.95 | 0.21, 18.0 | 0.32 |  |  |  |

^1^ Household asset score generated with principal component analysis (PCA) as was done in the original analysis of the National Nutrition Survey data (Nutrition Program, Department of Public Health, Ministry of Health, 2016) but restricted to households with a child <5 years old who participated in the anthropometry assessment. The included variables were 1) type of water source, shared sanitation facilities, toilet facilities, floor materials, roof materials, wall materials, cooking fuel, ownership of land, ownership of livestock, land of orchard, dry land, wet land, number of rooms, ownership of livestock, type of livestock (cattle/buffalo/yaks, pigs, horses, goats/sheep, poultry), sofa set, electric iron, bukhari, rice cooker, curry cooker, refrigerator, modern stove, water boiler, microwave oven, bicycle, tractor, power tiller, jewelry, motorbike/scooter, sechu gho/kira, family car, other vehicle, washing machine, sewing machine, television, VCR/VCD/DVD, grinding machine, wrist watch, and weaving tool.

^2^ Bhutan-specific definition of household access to an improved water source is piped water into the household only.

^3^ In the survey, mothers were asked if she adds anything to the water to make it safer to drink.

^4^ Food insecurity was one or more affirmative answers to the series of food security questions, represented as a composite variable of food insecurity that household experienced any out of four cases in the last month: (1) worry about not enough foods, (2) eat only rice/kharang/flour, (3) eat a smaller amount/skip meals at any meal time, and (4) eat fewer meals in a day.

^5^ Food consumption score (FCS) was based household dietary diversity, food frequency, and relative nutritional importance of different food groups in the past 7 days (World Food Programme, 2008).
