# Supplementary material for: Nutritional status and risk factors for stunting in preschool children in Bhutan
Source: Matern Child Nutr. 2018 Nov 9;14(Suppl 4):e12653. doi: 10.1111/mcn.12653 (PMC6587444; doi:10.1111/mcn.12653)
Supplement: Supplementary file 11 — Table S8. Child nutritional status, morbidity, and dietary practices in children with and without oedema in National Nutrition Survey 20151 [file MCN-14-e12653-s011.docx]

**Supplemental Table 8. Child nutritional status, morbidity, and dietary practices in children with and without edema in National Nutrition Survey 2015^1^**

|  | Children with edema (maximum n = 26) | Children without edema (maximum n = 1,480) | P-value^2^ |
| --- | --- | --- | --- |
|  | Mean (SD) | Mean (SD) |  |
| Growth |  |  |  |
| HAZ | -0.71 (1.26) | -0.87 (1.63) | 0.63 |
| WHZ | -0.06 (0.87) | 0.12 (1.04) | 0.38 |
| WAZ | -0.42 (0.95) | -0.42 (1.24) | 0.99 |
|  |  |  |  |
| Undernutrition | % | % |  |
| Stunting | 20.0 | 21.6 | 0.84 |
| Wasting | 0.0 | 2.9 | 0.39 |
| Underweight | 4.0 | 8.6 | 0.41 |
|  |  |  |  |
| Infectious morbidity | % | % |  |
| Fever | 26.1 | 21.7 | 0.61 |
| ARI | 0.0 | 1.2 | 0.61 |
| Diarrhea | 17.4 | 6.9 | 0.05 |
|  |  |  |  |
| Diet | % | % |  |
| Minimum dietary frequency | 55.0 | 45.7 | 0.41 |
| Minimum acceptable diet for all breastfed children | 3.9 | 3.0 | 0.81 |

HAZ: Height-for-age z-score; WAZ: Weight-for-age z-score; WHZ: Weight-for-height z-score;

^1^Edema was assessed holding one foot in each hand and applying pressure with the thumbs to the top of each foot for three seconds

^2^P-values were tested for Student t-test for continuous variables and chi2 test for categorical variables
